# Longitudinal and Cross-Sectional Sampling of Serpentovirus (Nidovirus) Infection in Captive Snakes Reveals High Prevalence, Persistent Infection, and Increased Mortality in Pythons and Divergent Serpentovirus Infection in Boas and Colubrids

**DOI:** 10.3389/fvets.2019.00338

**Published:** 2019-10-03

**Authors:** Laura L. Hoon-Hanks, Robert J. Ossiboff, Pia Bartolini, Susan B. Fogelson, Sean M. Perry, Anke C. Stöhr, Shaun T. Cross, James F. X. Wellehan, Elliott R. Jacobson, Edward J. Dubovi, Mark D. Stenglein

**Affiliations:** ^1^Department of Microbiology, Immunology, and Pathology, College of Veterinary Medicine and Biomedical Sciences, Colorado State University, Fort Collins, CO, United States; ^2^Department of Comparative Diagnostic and Population Medicine, College of Veterinary Medicine, University of Florida, Gainesville, FL, United States; ^3^Terrestrial & Arboreal, LLC, Melrose, FL, United States; ^4^Fishhead Labs LLC, Stuart, FL, United States; ^5^Department of Veterinary Clinical Sciences, School of Veterinary Medicine, Louisiana State University, Baton Rouge, LA, United States; ^6^Animal Health Diagnostic Center, College of Veterinary Medicine, Cornell University, Ithaca, NY, United States

**Keywords:** captive, epidemiology, nidovirus, pythons, respiratory disease, serpentovirus, snake

## Abstract

The aim of this study of serpentovirus infection in captive snakes was to assess the susceptibility of different types of snakes to infection and disease, to survey viral genetic diversity, and to evaluate management practices that may limit infection and disease. Antemortem oral swabs were collected from 639 snakes from 12 US collections, including 62 species, 28 genera, and 6 families: Pythonidae (*N* = 414 snakes; pythons were overrepresented in the sample population), Boidae (79), Colubridae (116), Lamprophiidae (4), Elapidae (12), and Viperidae (14). Infection was more common in pythons (38%; 95% CI: 33.1–42.4%), and in boas (10%; 95% CI: 5.2–18.7%) than in colubrids (0.9%, 95% CI: <0.01–4.7%); infection was not detected in other snake families (lamprophiids 0/4, 95% CI: 0–49%; elapids 0/12, 95% CI: 0–24.2%; and vipers 0/14, 95% CI: 0–21.5%), but more of these snakes need to be tested to confirm these findings. Clinical signs of respiratory disease were common in infected pythons (85 of 144). Respiratory signs were only observed in 1 of 8 infected boas and were absent in the single infected colubrid. Divergent serpentoviruses were detected in pythons, boas, and colubrids, suggesting that different serpentoviruses might vary in their ability to infect snakes of different families. Older snakes were more likely to be infected than younger snakes (*p*-value < 0.001) but males and females were equally likely to be infected (female prevalence: 23.4%, 95% CI 18.7–28.9%; male prevalence: 23.5%, 95% CI 18–30.1%; *p*-value = 0.144). Neither age (*p*-value = 0.32) nor sex (*p*-value = 0.06) was statistically associated with disease severity. Longitudinal sampling of pythons in a single collection over 28 months revealed serpentovirus infection is persistent, and viral clearance was not observed. In this collection, infection was associated with significantly increased rates of mortality (*p*-value = 0.001) with death of 75% of infected pythons and no uninfected pythons over this period. Offspring of infected parents were followed: vertical transmission either does not occur or occurs with a much lower efficiency than horizontal transmission. Overall, these findings confirm that serpentoviruses pose a significant threat to the health of captive python populations and can cause infection in boa and colubrid species.

## Introduction

The order *Nidovirales* historically included viruses infecting mammals, birds, fish, crustaceans, and insects. With the increasing availability and affordability of metagenomic sequencing, this order has seen marked expansion in the recent years [e.g. (1–3)]. A distinct clade of nidoviruses now classified as belonging to the suborder *Tornidovirineae* family *Tobaniviridae* began with the discovery of novel nidoviruses in pythons, marking the first example of nidoviruses in reptilian hosts (4–6). Since this initial discovery, related nidoviruses have been discovered in additional snake species, lizards, turtles, cattle, and snake-associated nematodes (1, 2, 7–11). Several of these viruses have been associated with significant disease, particularly respiratory disease, highlighting this virus family as a source of potential emerging pathogens that warrant additional investigation (4–8, 10, 11). With recent taxonomic reclassification of the order *Nidovirales*, reptile-associated nidoviruses are classified within the *Serpentovirinae* subfamily and will be referred following to as serpentoviruses (12).

Snake-associated (ophidian) serpentoviruses were discovered in 2014 and were indirectly linked to respiratory disease in pythons (4–6, 10). Subsequent experimental infections of ball pythons confirmed a causal relationship between serpentovirus infection and respiratory disease, emphasizing the importance of these emerging viruses in veterinary medicine (13). Experimentally infected pythons developed antemortem hyperemia of the choanal and oral mucosa with or without mucosal hemorrhages, abundant oral mucus secretion, excessive swallowing, increased respiratory effort and rate, open-mouthed breathing, and anorexia. Postmortem findings included chronic-active mucinous and proliferative rhinitis, stomatitis, glossitis, tracheitis, esophagitis, and interstitial pneumonia (13). Related viruses have been detected in snake species, with or without evidence of respiratory disease, spanning multiple families within the *Serpentes* suborder throughout North America, Europe, and Asia. These include *Pythonidae* species [ball python (*Python regius*), Indian python (*Python molurus*), Burmese python (*Python bivittatus*), green tree python (*Morelia viridis*), and carpet python (*Morelia spilota*)]; *Boidae* species [boa constrictor (*Boa constrictor*)]; *Colubridae* species [Pope's keelback (*Hebius popei*), red-banded snake (*Lycodon rufozonatus*), and Mandarin rat snake (*Euprepiophis mandarinus*)]; and *Homalopsidae* species [Chinese water snake (*Myrrophis chinensis*)] (2, 4–6, 9, 10, 14). Despite this growing list of susceptible hosts, the prevalence and clinical significance of serpentovirus infection in most snakes remains unknown, and no published study has tested snakes in the families *Viperidae* or *Elapidae*, two large families that include the majority of venomous snake species. Therefore, despite mounting evidence that serpentoviruses are common and potentially significant pathogens of snakes, the extent of species susceptibility to infection and disease remains poorly understood.

The purpose of this study was to evaluate the epidemiology of serpentovirus infection in captive snakes. Mixed captive snake collections with both known and unknown serpentovirus infection status were selected. The collections included snakes from the major snake families: *Pythonidae, Boidae, Colubridae, Lamprophiidae, Viperidae*, and *Elapidae*. Data from individual snakes within each collection was collected to assess correlations between species, age, sex, and clinical signs with serpentovirus status. Furthermore, sequencing data was generated to investigate serpentovirus genetic diversity and whether strains clustered by collection, species, and disease state. Longitudinal sampling was performed to determine persistence and progression of infection within a collection and evaluate the potential for viral clearance and vertical transmission.

## Materials and Methods

### Ethical Statement

All samples were considered non-invasive clinical samples collected by veterinarians or veterinary technicians from client-owned animals for the purpose of diagnostic testing for snake pathogens; an ethical review was not performed for this reason.

### Snake Collections/Populations

Eleven collections (A–K) were tested, either in entirety (all the snakes were tested) or partially (a subset of snakes). A twelfth, collective group (L) included snakes from a variety of sources that were individually submitted by veterinarians or owners following suspicion of serpentovirus-associated respiratory disease. One collection was sampled longitudinally (collection A) while the remaining collections were sampled once. Species, age, sex, clinical signs, and a respiratory score (see below) were recorded for each snake, when available. Snakes were recorded as either the species or subspecies depending on the information provided by the owner. If known, exact ages were recorded; in cases where only the age category was noted a standard age was provided (hatchling: ~1 month [0.1 year] and juvenile: 6 months [0.5 year]; adults were not estimated). If an age or age category was not provided, these snakes were not included in analyses where age was a variable. Sex was recorded as male, female, or unknown. Following evaluation of snakes and discussion with owners, veterinarians or veterinary technicians provided a respiratory score based on a specific rubric: 0—no respiratory signs; 1—mild respiratory signs (e.g., increased mucus in the oral cavity or hyperemia of the oral mucosa but otherwise acting normally); 2—moderate respiratory signs (e.g., wheezing, coughing); and 3—severe respiratory signs (e.g., open-mouthed breathing, respiratory distress). Summary data for each collection can be found in Supplemental Table S1.

### Longitudinal Sampling

Collection A was tested longitudinally following an outbreak of serpentovirus-associated respiratory disease. The first pythons in this collection were tested by in 2015 by PCR and metagenomic sequencing in the Stenglein laboratory (see below), at which point serpentovirus was determined to be established in the collection, but an overt outbreak had not occurred. In December 2015, a group of green tree pythons were purchased from another state. The purchased green tree pythons were acquired from the seller in July 2016, by which time approximately 9 of the purchased snakes had died of undiagnosed respiratory disease. None of the deceased snakes underwent postmortem examination by the sellers during this time (December 2015–July 2016), but two snake carcasses (A98 and A99) were frozen. Upon acquisition of the remaining live snakes, a quarantine area was established. The newly arriving snakes were placed in a large room that was partially separated from the main collection of pythons (i.e., quarantined pythons were housed in one room and the main collection pythons were housed in another room of the house, which was separated by space but not physical barriers); all venomous snakes were housed in rooms separated by walls and doors from the quarantined and main collection pythons. Two weeks into the quarantine period, purchased snakes began to show signs of respiratory disease; snakes began to die at 3 weeks into quarantine. Shortly after, pythons in the main collection also exhibited signs of respiratory disease. At this point, all pythons in the collection were sampled and any snakes found to be serpentovirus-positive, or had significant risk of exposure to serpentovirus (e.g., were part of the purchased collection, were housed in the quarantine area, were exhibiting signs of respiratory disease but initial testing yielded negative PCR results, or had any direct or indirect contact with purchased snakes) were separated from the main collection and placed in a separate building away from the house where more rigorous quarantine and disinfection measures were undertaken. These included separate tools and supplies that remained in quarantine, cleaning tools and surfaces with a quaternary ammonium compound (Simple Green d Pro 3 Plus), outer clothing and footwear specific for the quarantine area, only one designated caretaker for infected snakes, showering directly after exiting the quarantine area, and air filters placed on all vents leading out of the quarantine building. All pythons within the collection had continued testing over the course of 28 months at approximately 4-month intervals. Furthermore, the two snake carcasses that were frozen by the seller were acquired and postmortem oral swabs were collected. In contrast to the sampling of the python species within the collection, only a subset of venomous snakes (12 elapids and 13 vipers) were tested and snakes were only tested once.

During the 28 months, a serpentovirus-positive male and female green tree python were bred in the quarantine area, resulting in a clutch of eggs. The eggs were removed from the parents and artificially incubated in the main collection area. Egg surfaces were cleaned by exposure to UV light (Zoo Med brand 5.0 light) for 1 min, 3–4 inches away. Following hatching, eggs were frozen and egg shells/remaining contents were submitted for sampling, along with choanal swabs from the hatchlings. Hatchlings were sampled at approximately 4-month intervals for 20 months.

Similarly, a serpentovirus-positive male and female jungle carpet python (*Morelia spilota cheynei*) were bred in quarantine, resulting in a clutch of eggs. The eggs were removed from the parents and artificially incubated in the quarantine area. Egg surfaces were briefly cleaned with a quaternary ammonium compound. Upon hatching, offspring were removed from quarantine and placed in a separate room away from both serpentovirus-positive snakes and the main collection. Eggs were tested following hatching and offspring were tested twice during a 6-month interval.

### Postmortem Examination

A necropsy was performed on a subset of snakes from collection A, H, and L that died during the study. Fresh frozen and formalin-fixed tissues were collected. Formalin-fixed tissues were processed routinely and histopathology was performed on an a select few of these cases, as previously described (13).

### Snake Sampling

#### Swabs

**Figure 1:**
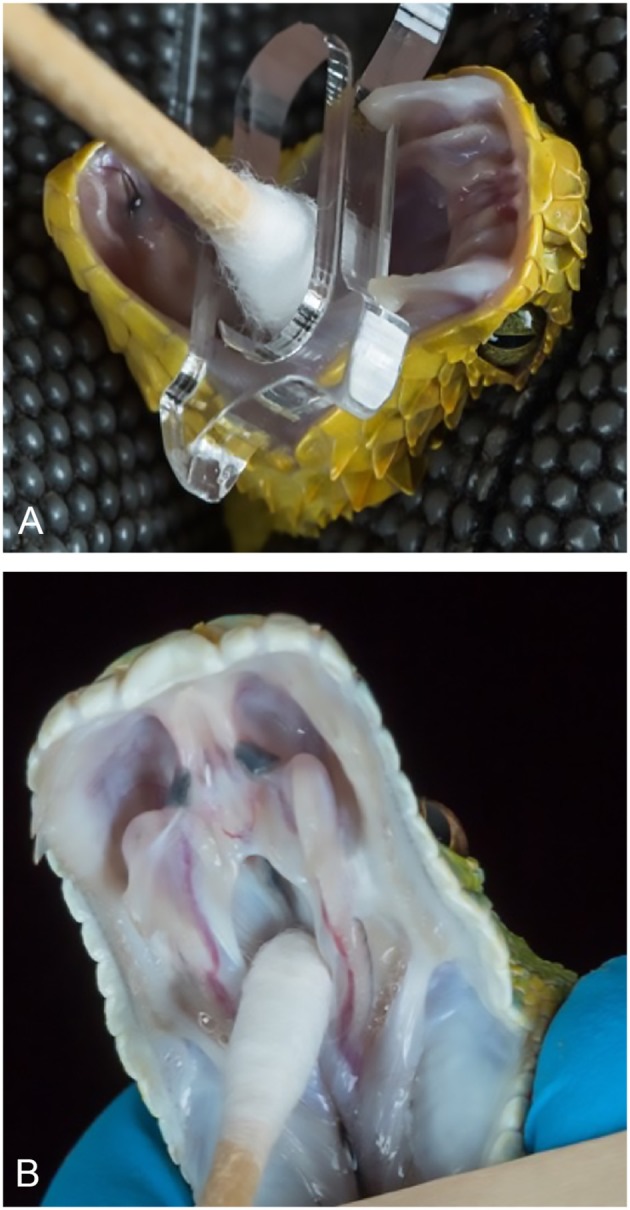
Oral (top) and choanal (bottom) swabbing of captive snakes. Variable bush viper *(Atheris squamigera)* **(A)**. Green tree python (*Morelia viridis*) **(B)**. Photos courtesy of Greg Lepara.

All antemortem samples were collected by a veterinarian or certified veterinary technician. Swabs from the choana or oral cavity were collected from each individual snake using cotton- or rayon-tipped swabs (Figure 1). Dry swabs were then placed in either a 1.5 ml Eppendorf tube or 2 ml screw-cap conical tube and frozen at −20°C for 1–14 days. Samples were then shipped overnight on ice to the Stenglein laboratory. At the time of arrival, swabs were suspended in 1 ml of brain heart infusion (BHI) broth (Becton Dickinson) and vortexed prior to storage at −80°C.

#### Tissues

Samples from post mortem analysis of collection A, H, and L (see above) were submitted as fresh-frozen tissues (−20 or −80°C). Lung, trachea, and esophagus were pooled or submitted as individual samples. Additionally, to assess vertical transmission in Collection A, eggs collected post-hatching were submitted frozen (−20°C). Tissue and egg samples were stored at −80°C upon arrival to the Stenglein laboratory.

### RNA Extraction

#### Swabs

Viral RNA was extracted from swabs in BHI using Zymo Research viral RNA kit with either individual columns or in a 96-well plate. Approximately 200 μl of BHI was processed according to the manufacturer's instructions. RNA was eluted in 30 μl of RNase/DNase-free water. Extracted RNA was stored at −80°C.

#### Tissues

Total RNA was extracted from tissues and pooled eggs (shell and remaining contents; 4 eggs per pool) using a combination of TRIzol (Ambion Life Technologies) with RNA clean and concentrator columns (CC-5; Zymo Research) as previously described (13). Extracted RNA was stored at −80°C.

### Viral RNA Detection

RNA extracted from snake samples was tested for serpentovirus RNA multiple times by independent methods. First, a hemi-nested polymerase chain reaction (PCR) was performed. Round 1, a reverse transcription quantitative PCR (RT-qPCR), was performed using Luna Universal One-Step RT-qPCR kit (New England BioLabs). Twelve microliter reactions included a final concentration of 1x Luna Universal One-Step Reaction Mix, 1x Luna WarmStart RT Enzyme Mix, and 0.3 μM of each degenerate serpentovirus primer (BarniPVTF and BarniGGTR; Table 1) mixed with 4 μl of RNA template. Reaction mixtures were run in a Roche LightCycler 480 II with the following cycle parameters: 55°C for 10 min; 95°C for 1 min; 95°C for 10 s and 60°C for 30 s with 45 cycles; and a melting curve (95°C for 5 s; 65°C for 1 min; ramp to 97°C with a rate of 0.11°C/s; and 40°C for 30 s). A no-template water control and positive RNA control from previous experiments with serpentovirus positive snakes (13) were included with each plate. Round 2, a qPCR, was performed using Luna Universal qPCR Master Mix. Twelve microliter reactions included a final concentration of 1x Luna Universal Master Mix and 0.3 μM of each degenerate serpentovirus primer (BarniPVTF and BarniDYTR; Table 1) mixed with 5 μl of PCR product from round 1 diluted 1:10 in water. Reaction mixtures were run with the following cycle parameters: 95°C for 1 min; 95°C for 15 s and 60°C for 30 s with 45 cycles; and a melting curve. Both round 1 and round 2 PCR products were run on a 1.5% agarose gel with ethidium bromide for confirmation of amplification and assessment of amplicon size.

**Table 1:** Primer sets targeting snake serpentoviruses.

**Primer**	**F/R**	**Sequence (**5^′^**-3**^′^**)**	**Target**	**Pair**	**Amplicon (bp)**
BarniPVTF (MDS-1222)	F	GAGCACTCCACAARCCAGTCAC	RdRp	BarniGGTR	353
				BarniDYTR	185
BarniGGTR (MDS-1224)	R	KGCATCRCCRCTACTTGTGCCTCC	–	–	–
BarniDYTR (MDS-1223)	R	RCTRCGGTCGCATTTCGTRTARTC	–	–	–
MDS-1529	F	GCAGCACCAGACAACTTCAT	ORF1B/RdRp	MDS-1530	536
				BarniDYTR	385
MDS-1530	R	TTGTACAGWGTGTTGGCGAA	–	–	–

*F, forward; R, reverse; RdRp, RNA-dependent RNA polymerase; ORF1B, open reading frame 1B; Bp, basepairs*.

RNA from all positive samples from the first hemi-nested PCR was re-tested by a second PCR protocol to confirm results. Reverse transcription from RNA to complementary DNA (cDNA) was performed using random priming as previously described (13). The heminested PCR described above was then repeated using the same primers (round 1: Barni PVTF and BarniGGTR; round 2: BarniPVTF and BarniDYTR) but utilizing the Luna Universal qPCR Master Mix reaction parameters and cycle conditions for both rounds.

A subset of samples with inconclusive PCR results were selected for an additional PCR using the internal primer set (BarniPVTF and BarniDYTR) in a non-nested reaction; this primer set was found to provide increased sensitivity for more divergent serpentoviruses when used independently of the outer primer set. PCR was performed as described above, beginning with diluted cDNA as template. Cycle parameters were altered as follows: 95°C for 1 min; 95°C for 15 s, 46°C for 20 s, and 60°C for 20 s with 45 cycles; and a melting curve.

To confirm previous positive results were not the outcome of PCR contamination during round 1 or round 2 of the hemi-nested reactions and to achieve a longer amplicon sequence for phylogenetic analysis, new primers were designed that flanked the previous hemi-nested set using partial or full-length ophidian serpentovirus sequences available in this laboratory from python hosts (MDS-1529 and MDS-1530; Table 1). Positive samples were tested as previously described using the new primers and beginning with random-primed cDNA as template. Cycle parameters were as follows: 95°C for 1 min; 95°C for 15 s and 60°C for 30 s with 45 cycles; and a melting curve.

### Sanger Sequencing

Samples that were positive by qPCR and demonstrated the correct amplicon size by gel electrophoresis were Sanger sequenced. Samples were submitted to GENEWIZ (San Diego, California) as premixed samples with the appropriate forward primer (BarniPVTF or MDS-1529); sequencing reactions with the reverse primer were not performed.

### Metagenomic Sequencing

A subset of samples with inconclusive PCR results were targeted for shotgun metagenomic sequencing (Supplemental Table S2). RNA libraries were generated using the Kapa RNA HyperPrep Kit (Kapa Biosystems) according to the manufacturer's instructions with an input concentration of approximately 100 ng of RNA and 8 rounds of amplification. Kapa Dual-Indexed Adapter Kit Illumina Platform (Kapa Biosystems) was used for adapter ligation and barcoding. Equivalent masses of DNA from each sample were pooled and libraries were size selected (200–600 bp, including Illumina adapters) by gel electrophoresis on a 2% agarose gel. Gel extraction was performed using the Zymo Gel DNA Recovery Kit (Zymo Research) according the manufacturer's instructions. Library quantification was performed with the Kapa Biosystems Illumina library quantification kit according the manufacturer's instructions. Dual indexed, single-end 1 × 75 or 1 × 150 sequencing was performed on an Illumina NextSeq 500 instrument with a NextSeq 500/550 High Output Kit v2 (75 cycles) or Mid Output v2 (150 cycles), respectively. Sequencing analysis was performed as previously described (13).

Sequences were deposited in the GenBank database and short read archive (SRA). Short sequences of ORF1B: GenBank MN117077-MN117130. Partial or full-length genomic sequences generated by metagenomic sequencing: Genbank MN161558-MN161572. Raw data from metagenomic sequencing: BioProject PRJNA555716, BioSample SAMN12358419-SAMN12358456, SRA SRR9921013-SRR9921050.

### Data Analysis

#### Statistical Analyses

Overall prevalence with 95% confidence intervals (CI) was assessed within each snake family (*Pythonidae, Boidae, Colubridae, Lamprophiidae, Elapidae, Viperidae*). The association between infection status and age, sex, respiratory score, and phylogenetic family were evaluated by a binary logistic regression. Age and sex were also independently compared to the respiratory score (0, 1, 2, 3) by a Kruskal-Wallis test and Mann-Whitney *U* test, respectivley.

#### Phylogenetic Analyses

Sequences generated by either Sanger sequencing or metagenomic sequencing were utilized for phylogenetic analysis. Sequences spanning a 467 bp region of ORF1B, including the RNA-dependent RNA polymerase, were aligned using MAFFT software with default parameters (E-INS-i algorithm, 200PAM/*k* = 2 scoring matrix, gap open penalty of 3, and offset value of 0) in Geneious 11.0.4 (15, 16). A phylogenetic tree was generated using PhyML in Geneious with Hasegawa-Kishino-Yano (HKY, nst = 2) substitution model, with 1000 bootstrap replicates, an estimated transition/transversion rate, a fixed proportion of invariable sites (0), 4 substitution rate categories, and a fixed gamma distribution parameter (1, 17, 18).

## Results

### Prevalence

**Figure 2:**
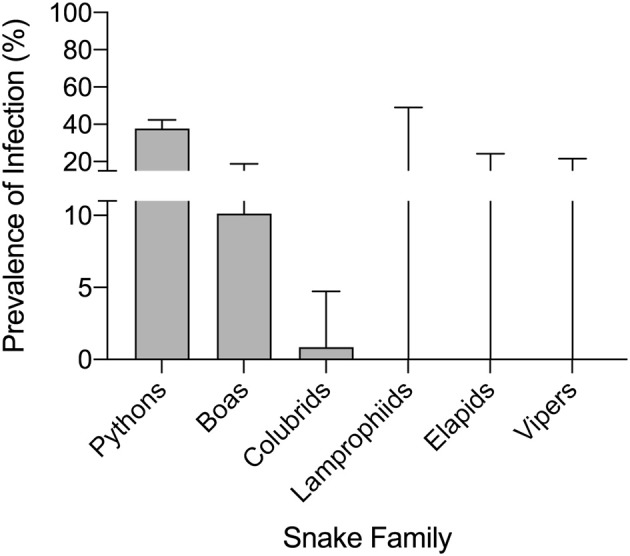
Captive pythons in some collections have a high prevalence of serpentovirus infection. A significant association was observed between infection status and snake family (*p*-value ≤ 0.001). Prevalence of serpentovirus in snakes of each family: pythons 37.7% (156 infected/414 total snakes tested; 95% confidence interval [CI]: 33.1–42.4%), boas 10.1% (8/79; 95% CI: 5.2–18.7%), colubrids 0.9% (1/116; 95% CI: <0.01–4.7%); infection was not detected in lamprophiids (0/4; 95% CI: 0–49%), elapids (0/12; 95% CI: 0–24.2%), or vipers (0/14; 95% CI: 0–21.5%). Bars indicate 95% CI.

Serpentovirus infection was detected in all snake collections except collection J, and prevalence within each serpentovirus-positive collection ranged from 5 to 100% (Supplemental Table S1). In total, 165 snakes out of 639 were found to be positive for serpentovirus (26%). Prevalence among pythons was markedly higher (37.7%; 156 infected/414 total; 95% CI 33.1–42.4%) compared to boas (10.1%; 8/79; 95% CI: 5.2–18.7%) and colubrids (<1%; 1/116; 95% CI: <0.01–4.7%); infection was not detected in lamprophiids (0/4; 95% CI: 0–49%), elapids (0/12; 95% CI: 0–24.2%), or vipers (0/14; 95 CI: 0–21.5%; Figure 2). Prevalence in individual species ranged from 0 to 100% (Table 2). A significant association was observed between infection status and snake family (*p*-value ≤ 0.001).

**Table 2:** Prevalence of serpentovirus by snake species, family, and total.

**Common name**	**Scientific name**	**Total # tested**	**# Positive**	**Positive (%)**
**Pythons (*****Pythonidae*** **family)**				
Children's python	*Antaresia childreni*	1	0	0%
Spotted python	*Antaresia maculosa*	3	0	0%
Anthill python	*Antaresia perthensis*	2	1	50%
Stimson's python	*Antaresia stimsoni*	1	1	100%
Black-Headed python	*Aspidites melanocephalus*	2	0	0%
Woma python	*Aspidites ramsayi*	3	1	33%
Bismarck ring python	*Bothrochilus boa*	2	0	0%
Savu python	*Liasis mackloti*	4	0	0%
Olive python	*Liasis olivaceus*	3	0	0%
Reticulated python	*Malayopython reticulatus*	14	6	43%
Rough scaled python	*Morelia carinata*	2	2	100%
Carpet python	*Morelia spilota*	13	8	62%
Jungle carpet python	*Morelia spilota cheynei*	8	6	75%
Inland carpet python	*Morelia spilota metcalfei*	2	2	100%
Diamond python	*Morelia spilota spilota*	4	4	100%
Green tree python	*Morelia viridis*	120	91	76%
Angolan python	*Python anchietae*	6	1	17%
Burmese python	*Python bivittatus*	2	0	0%
Borneo python	*Python breitensteini*	20	2	10%
Blood python	*Python brongersmai*	45	16	36%
Sumatran python	*Python curtus*	20	7	35%
Indian rock python	*Python molurus*	1	1	100%
Ball python	*Python regius*	136	7	5%
	**Totals**	**414**	**156**	**37.7%**
**Boids (*****Boidae family*****)**				
Dumeril's boa	*Acrantophis dumerili*	2	1	50%
Boa constrictor	*Boa contrictor*	16	0	0%
Puerto Rican boa	*Chilabothrus inornatus*	1	1	100%
Emerald tree boa	*Corallus caninus*	29	5	17%
Amazon tree boa	*Corallus hortulanus*	2	1	50%
Brazilian rainbow boa	*Epicrates cenchria*	5	0	0%
Kenyan sand boa	*Gongylophis colubrinus*	11	0	0%
West African sand boa	*Gongylophis muelleri*	10	0	0%
Rosy boa	*Lichanura trivirgata*	3	0	0%
	**Totals**	**79**	**8**	**10.1%**
**Colubrids (*****Colubridae*** **family)**				
Western hognose	*Heterodon nasicus*	7	0	0%
CA kingsnake	*Lampropeltis getula californiae*	16	0	0%
Nuevo Leon kingsnake	*Lampropeltis exicana thayeri*	17	0	0%
AZ mountain kingsnake	*Lampropeltis pyromelana*	9	0	0%
Milksnake	*Lampropeltis triangulum*	1	0	0%
LA milksnake	*Lampropeltis t. amaura*	2	0	0%
Pueblan milksnake	*Lampropeltis t. campbelli*	3	0	0%
Honduran milksnake	*Lampropeltis t. hondurensis*	4	1	25%
Nelson's milksnake	*Lampropeltis t. nelsoni*	1	0	0%
Sinaloan milksnake	*Lampropeltis t. sinaloae*	6	0	0%
Baja CA mountain kingsnake	*Lampropeltis zonata algama*	3	0	0%
Tricolor hognose	*Lystrophis pulcher*	7	0	0%
Cornsnake	*Pantherophis guttatus*	34	0	0%
Bullsnake	*Pituophis catenifer sayi*	2	0	0%
Cape gopher snake	*Pituophis vertebralis*	4	0	0%
	**Totals**	**116**	**1**	**0.9%**
**Lamprophiids (*****Lamprophiidae*** **family)**				
African house snake	*Boaedon filiginosus*	4	0	0%
	**Totals**	**4**	**0**	**0%**
**Elapids (*****Elapidae*** **family)**				
Death adder	*Acanthophis rogosus*	1	0	0%
Angolan coral cobra	*Aspidelaps lubricus cowlesi*	1	0	0%
Shield-nosed cobra	*Aspidelaps scutatus*	1	0	0%
Blackbacked Jameson's mamba	*Dendroaspis jamesoni jamesoni*	3	0	0%
Black mamba	*Dendroaspis polylepis*	1	0	0%
Western green mamba	*Dendroaspis viridis*	3	0	0%
Eastern green mamba	*Dendroaspis angusticeps*	1	0	0%
Rinkhal's spitting cobra	*Hemachatus haemachatus*	1	0	0%
	**Totals**	**12**	**0**	**0%**
**Vipers (*****Viperidae*** **family)**				
Usambara eyelash bush viper	*Atheris ceratophora*	3	0	0%
Mexican nomad viper	*Atropoides nummifer*	1	0	0%
Speckled forest pit viper	*Bothriopsis taeniata*	4	0	0%
Brazilian lance-head pit viper	*Bothrops moojeni*	2	0	0%
Sumatran pit viper	*Trimeresurus sumatranus*	1	0	0%
Sri Lankan palm pit viper	*Trimeresurus trigonocephalus*	3	0	0%
	**Totals**	**14**	**0**	**0%**
	**Totals**	**639**	**165**	**25.8%**

In collections A and H, physical separation of infected snakes from the rest of the collection and strict sterility practices resulted in high prevalence of infection in quarantined snakes and low prevalence in the main collection. In these two collections, quarantined snakes were relocated to a separate building from the main collection that included its own separate air-flow/venting. Quarantine practices included designated individuals or days for managing quarantined snakes, shower-out procedures, quarantine-specific clothes, shoes, equipment, and instruments, one-way flow of bedding and feeder rodents, disposable glove changes between racks and hand sanitizer disinfection of gloves between breed rotations in racks, and disinfection of all surfaces and instruments with quaternary ammonium compound (Simple Green or F10) following use. In collection A, the prevalence of serpentovirus in quarantined snakes (A1–9 and 13–36) was 94% (31/33). Prevalence within the primary collection was 4%: only 1 of 25 pythons became positive (a green tree python; see “Long-Term Sequential Sampling”). In collection H, prevalence in quarantined snakes (H118–154) was 68.4% (26/38) and prevalence in the main collection (H1–117) was 4.3% (5/117). The quarantine practices for other collections were unknown or quarantine of infected snakes did not occur.

### Morbidity and Mortality

**Figure 3:**
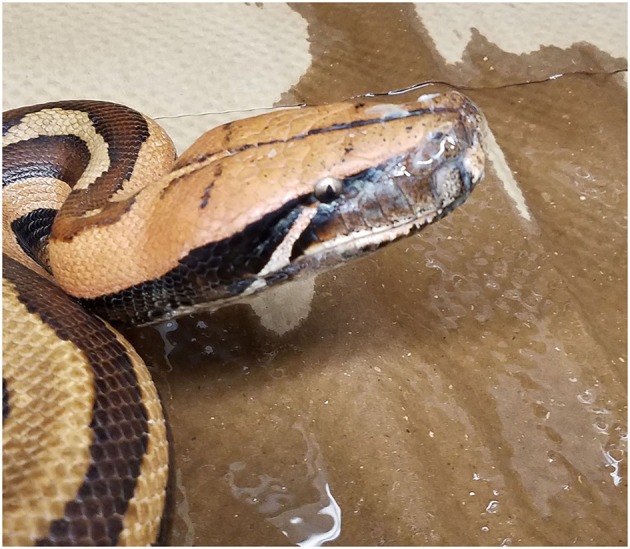
Excessive oral and nasal mucus production in a serpentovirus-positive snake. Blood python; Collection H.

**Figure 4:**
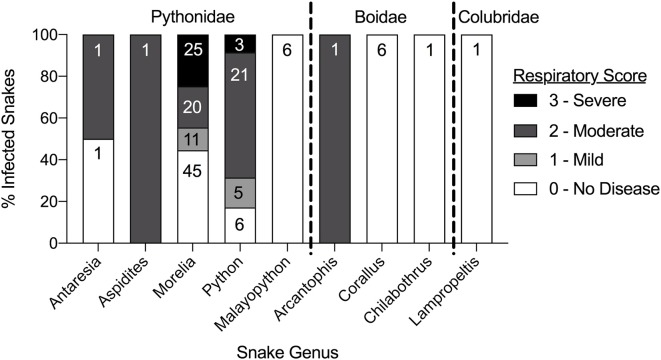
Clinical disease varies by individuals within each snake genus and amongst snake families. Comparison of the respiratory scores of individual serpentovirus-positive snakes within each snake genus. Data is represented as a stacked bar graph totaling 100% of infected snakes in each snake genus. Each respiratory score is represented as a percentage of the total. Values within each fraction of the total indicate the sample size (N).

Clinical signs noted at the time of sampling included increased oral hyperemia and increased oral and nasal mucus (respiratory score 1; Figure 3), wheezing and/or audible breathing (respiratory score 2), open-mouthed breathing or difficulty breathing (respiratory score 3), and other non-specific signs such as anorexia, inappropriate shedding, difficulty perching in arboreal snakes, and spectaculitis. Respiratory scores were compared between snakes in each genus with at least one positive result (Figure 4). Scores ranged from 0 (absent disease) to 3 (severe disease) within snakes of the *Pythonidae* family, including within each genus. In contrast, only 1 out of 8 snakes within the *Boidae* family had a non-zero respiratory score. Only one snake from the *Colubridae* family was found to be positive and had no apparent signs of disease (respiratory score of 0). Statistical comparisons were not performed due to the low number of snakes available in each genus other than *Morelia* and *Python*. Low numbers of snakes that were serpentovirus-negative also exhibited signs of respiratory disease (Supplemental Table S1).

**Figure 5:**
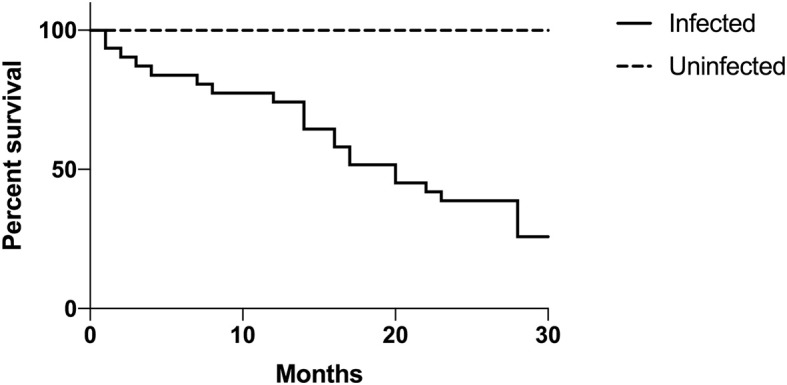
Infected snakes are statistically more likely to experience mortality than uninfected snakes. Longitudinal assessment of mortality between infected and uninfected snakes in collection A. Time is represented in months and begins at the onset of a serpentovirus outbreak (September 2016). Kaplan-Meier survival curve; *p*-value = 0.001.

Mortality rates were high in three examined collections. In collection A, the mortality rate of serpentovirus-infected snakes was 75% (30/40). Within 20 months of initial sampling, 50% of infected pythons in collection A had died, in comparison to none of the uninfected pythons, which were all still alive at the end of the study (Figure 5). In collection G, the mortality rate of infected snakes was 45% (9/20); euthanasia of some serpentovirus-uninfected snakes due to a *Cryptosporidium* outbreak, as well as the sale of uninfected snakes and loss to follow-up, precluded assessment of serpentovirus-uninfected snake mortality. In collection H, 5 infected snakes were found dead and 51 snakes with either confirmed serpentovirus-infection or contact with infected snakes were euthanized. The total loss in the collection was 36% (56/155); the mortality rate of uninfected snakes was 0%.

**Figure 6:**
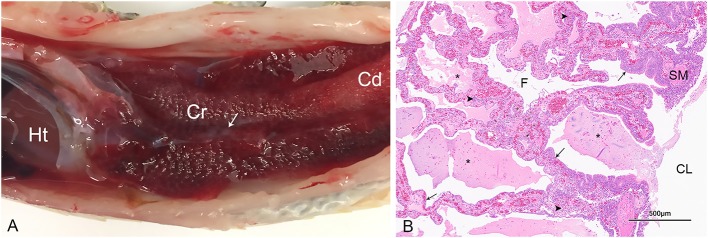
Gross and histologic pulmonary lesions in a serpentovirus-positive green tree python from collection A (snake A26). **(A)** The lungs are markedly thickened and dark red, especially in the cranial (Cr) lung compared to the caudal (Cd) lung. Small mucoid aggregates are present within the central lumen of the lung (arrow). The heart (Ht) is indicated for orientation. **(B)** Histopathology of the lung revealing proliferative and interstitial mucinous pneumonia characterized by excessive mucus, edema, and necrotic debris (asterisks) within the central lumen (CL) and faveoli (F), marked epithelial hypertrophy and hyperplasia lining faveoli (arrows), and mixed chronic-active inflammation within thickened faveolar septa (arrowheads). These lesions are consistent with serpentovirus infection. SM, smooth muscle. Hematoxylin and Eosin. 40× magnification.

A subset of snakes that were PCR positive for serpentovirus by oral swab and/or tissue pools (lung, trachea, esophagus) were assessed by gross, histopathologic, or complete postmortem evaluation from collections A (15 pythons), H (1 python), and L (7 pythons). Collection A included 11 green tree pythons (*Morelia viridis*), 2 carpet pythons (*Morelia spilota*), and 2 rough scaled pythons (*Morelia carinata*); a single blood python (*Python brongersmai*) was evaluated in collection H; and collection L included 4 green tree pythons, 1 rock python (Python molurus), 1 Stimson's python (*Antaresia stimsoni*), and 1 Dumeril's boa (*Acrantophis dumerili*). All pythons (total 23) exhibited evidence of respiratory disease consistent with serpentovirus infection as previously described (4–6, 10, 13). Findings included chronic-active and mucinous rhinitis, stomatitis, esophagitis, tracheitis, and proliferative interstitial pneumonia. Representative gross and histologic images are presented in Figure 6. Moderate to severe pneumonia was determined to be a primary contributor to the cause of death in most snakes (all except 4 green tree pythons) and these snakes exhibited a respiratory score of 2 (moderate) or 3 (severe) around the time of death. Secondary lesions detected by histopathology (*n* = 10) included bacterial and/or fungal colonization of the upper and lower respiratory tract and esophagus (*n* = 7), sepsis (*n* = 3), and systemic gout (likely associated with emaciation and dehydration; *n* = 1). Bacterial culture of the lung or liver from two snakes yielded heavy growth of *Pseudomonas aeruginosa* and moderate growth of *Escherichia coli, Providencia rettgeri*, and/or *Citrobacter* species. In the 4 green tree pythons with minimal to mild pneumonia, lesions in the upper respiratory tract (rhinitis, stomatitis, esophagitis, tracheitis) were more pronounced and were accompanied by evidence of emaciation, gout, or sepsis that was determined to be a likely cause of death. These snakes exhibited respiratory scores of 1 (mild) around the time of death.

### Age and Sex

**Figure 7:**
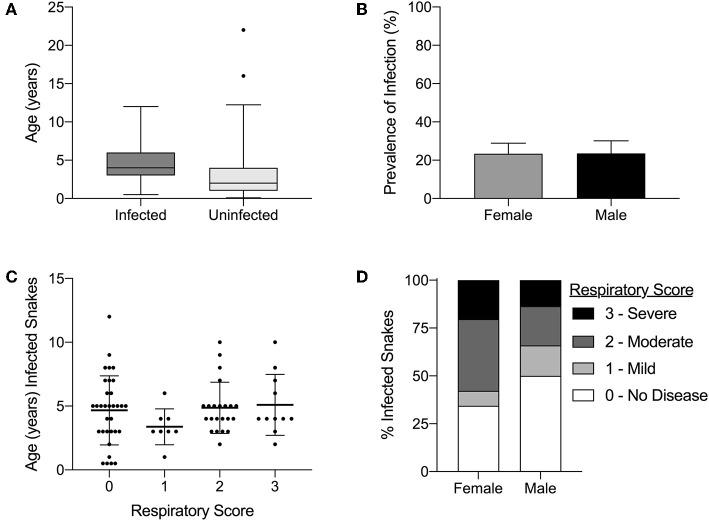
Serpentovirus infection is statistically associated with age but not with sex; clinical signs are not statistically associated with age or sex. **(A)** Comparison of ages between serpentovirus-infected and uninfected snakes. Average ages: 4.7 (infected) and 2.6 (uninfected). Binary logistic regression; *p*-value ≤ 0.001. Box and whisker plot 1–99 percentile. **(B)** Prevalence of infection in male and female snakes. Female prevalence: 23.4%, 95% CI 18.7–28.9%. Male prevalence: 23.5%, 95% CI 18–30.1%. Binary logistic regression; *p*-value = 0.144. **(C)** Comparison of respiratory score to age in serpentovirus-positive snakes. Kruskal-Wallis test (one-way ANOVA on ranks, non-parametric); *p*-value = 0.32. Scatter dot plot mean with standard deviation. **(D)** Comparison of respiratory score with sex in serpentovirus-positive snakes. Mann-Whitney *U* test; *p*-value = 0.06. Stacked bar graph representing the number of snakes with each respiratory score as a fraction of the total (100%).

Infection status was compared to age (range of 0.1–22 years) and sex (male or female). A statistically significant association was observed between age and infection status in snakes (*p*-value ≤ 0.001; Figure 7A). No significant association was observed between the sex and infection status (*p*-value = 0.144; Figure 7B). Neither age (Figure 7C) nor sex (Figure 7D) were statistically associated with respiratory score (*p*-value = 0.32 and *p*-value = 0.06, respectively).

### Phylogenetic Analysis

**Figure 8:**
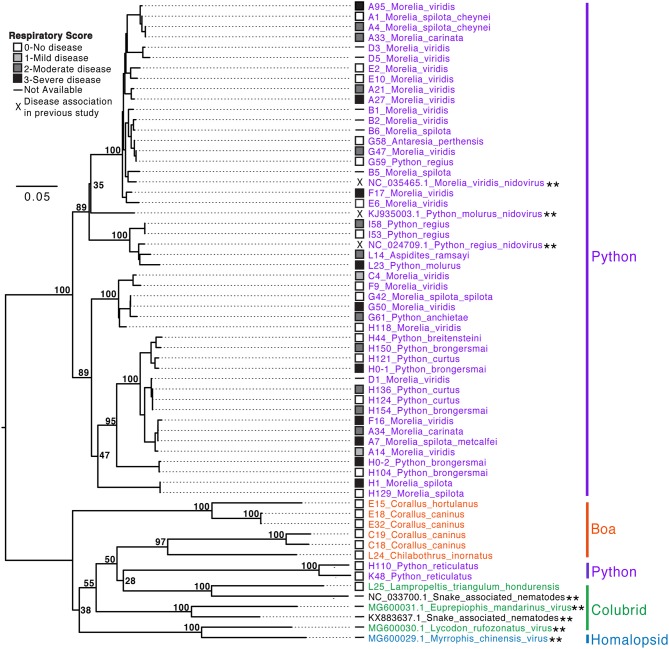
Serpentovirus phylogeny reveals implications regarding species susceptibility to infection and disease. Snake IDs are indicated by collection letter (A–L) and number followed by genus and species. Snake families are indicated to the right: Python (*Pythonidae*, purple), Boa (*Boidae*, orange), Colubrid (*Colubridae*, green), and Homalopsid (*Homalopsidae*, blue). Reference sequences identified in previous studies and available in GenBank (double asterisks: ^**^) are indicated by accession number followed by the genus and species of the host, except snake-associated nematode viruses in which the host species was unknown. Nucleotide sequences (467 basepairs; ORF1B) aligned and analyzed with PhyML with HKY85 substitution model and 1,000 bootstrap replicates; bootstrap values (%) included at major nodes. The tree was semi-subjectively rooted, this was partially based on protein alignments of ORF1B of serpentovirus reference sequences in GenBank and partially to highlight differences between the python-only clade and more divergent viral sequences of boas, colubrids, homalopsids, and reticulated pythons; a definitive monophyletic clade of these divergent viruses is poorly supported by bootstrap values and should not be interpreted as such. Short sequences of the ORF1B detected during the study and used to generate this phylogeny were deposited in GenBank (MN117077-MN117130).

Viral sequences were phylogenetically analyzed to assess overall genetic diversity, to reconstruct possible transmission events, and to test for associations between viral genotypes and host species or disease (Figure 8; Genbank accession numbers MN117077-MN117130).

Two serpentovirus groups were evident. One definitive clade, which accounted for most of the sequences generated in this study, contained sequences recovered from python species in the genera *Morelia, Python, Aspidites*, and *Antaresia* (Figure 8). This clade included the serpentoviruses previously associated with respiratory disease in pythons, such as ball python nidovirus (4–6, 10, 13). The sequences in this python-only clade shared >80% pairwise nucleotide identity in the aligned ORF1B region.

The other group contained sequences from boas (genera *Corallus* and *Chilabothrus*), a colubrid (genus *Lampropeltis)*, and reticulated pythons (genus *Malayopython)* in addition to sequences that were identified by metagenomic surveys of colubrid and homalopsid snakes in China (2). This second group was less well-supported phylogenetically and contained more overall genetic diversity than the python-only clade, with sequences sharing ≥57% pairwise nucleotide identity. These sequences were identified using metagenomic sequencing of samples from snakes for which PCR results had been negative or inconclusive (Supplemental Table S2).

Although the virus phylogeny did not form well-supported monophyletic clades by host, viruses with more similar sequences were generally found in snakes of the same species or genus. Exceptions to this included closely related viruses that infected pythons of multiple genera in the same collection. For example, virtually identical viruses (≥99.8% pairwise identity in the region used to make the tree) were recovered from three snakes of different genera from collection G: G47 (*Morelia viridis*), G58 (*Antaresia perthensis*), and G59 (*Python regius*). Similar examples included the viruses from snakes G42, G50, and G61, and those from L14 and L23. Genetically distinct serpentovirus sequences were recovered from all positive collections, except collection I.

The possibility that some viral genotypes were more pathogenic than others was also investigated. In the python-only clade, respiratory score ranged from 0 to 3 and an overt association between viral genotype and clinical disease was not evident. In contrast, respiratory scores were 0 for all boas, colubrids, and reticulated pythons outside the python-only clade. The only boa in this study that was documented to have clinical disease (L22, Dumeril's boa; respiratory score of 2) was not included in this phylogeny due to sequence length limitations (155 bp available compared to 467 bp for sequences in the phylogeny). Alignment of the short Dumeril's boa sequence revealed it to be slightly more related to the viruses in the python-only clade, with which it shared ~70–77% nucleotide identity, in contrast to 49–62% identity to viruses derived from boas, colubrids, and reticulated pythons.

Partial or complete genome sequences derived from metagenomic sequencing were globally aligned to ball python nidovirus (BPNV; NC_024790.1) and percent nucleotide identity was assessed (Supplemental Table S2). Python serpentoviruses (A93–95, F17, H0–1,−2, L1, 3, 4, 8, 14) ranged from 62 to 94% nucleotide identity; boa serpentoviruses (C18–19) ranged from 31 to 43% nucleotide identity; the colubrid serpentovirus (L25) exhibited 30% nucleotide identity; the reticulated python serpentovirus (K48) exhibited 31% nucleotide identity. These new serpentovirus genomes had similar overall genome structures to that of BPNV at the 5' end: two large overlapping open reading frames, ORF1A and ORF1B, separated by a ribosomal frameshift signal (AAAAAC) that together encode a large polyprotein of non-structural proteins. These large ORFs were immediately followed by a spike protein gene (ORF2). The 3' end of the genomes of the viruses in the python-only clade were similarly organized to BPNV with five predicted ORFs encoding: a transmembrane protein, matrix protein, nucleoprotein, and 2 additional predicted transmembrane proteins. The other serpentoviruses outside the python-only clade for which near-complete genomes were obtained had more variable 3′ end gene content, but all had predicted matrix and nucleoprotein genes flanked by variable numbers of genes encoding proteins with predicted transmembrane domains.

### Co-infection

Serpentovirus co-infection in a blood python from collection H (snake H0) was detected. One complete coding genome (H0–1) and one nearly complete coding genome (H0–2) were generated from sequencing reads (Supplemental Table S2). These sequences shared approximately 71% global nucleotide identity. When a short nucleotide sequence from each H0 serpentovirus was compared to other snakes in the collection (Figure 8), H0–1 shared >99% nucleotide identity to H121 and only 90% to H0–2 and H104, respectively; H0–2 shared >99% identity to H104. Possible coinfections were also detected in two other snakes (H136 and L21) in which low numbers of unique serpentovirus reads detected by metagenomic sequencing did not align to the primary assembled serpentovirus genome.

### Long-Term Sequential Sampling of Collection A

**Figure 9:**
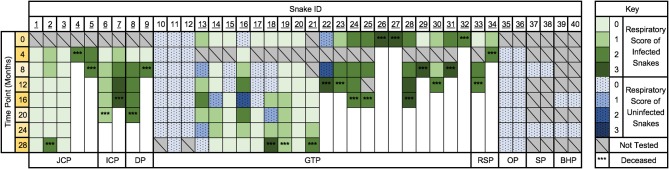
The progression of serpentovirus infection and disease within a single collection (A) over 28 months. Time points begin September 2016 (0 months) and end December 2018 (28 months). Green indicates a serpentovirus-positive result (infected). Blue indicates a serpentovirus-negative result (uninfected). Shades of blue and green indicate respiratory score at the time of sampling. ^***^Indicate the approximate time of death. Snakes in quarantine are indicated by underlining of the snake ID number. JCP, jungle carpet python (*Morelia spilota cheynei*). ICP, inland carpet python (*Morelia spilota metcalfei*). GTP, green tree python (*Morelia viridis*). RSP, rough-scaled python (*Morelia carinata*). OP, olive python (*Liasis olivaceus*). SP, savu python (*Liasis mackloti savuensis*). BHP, blackhead python (*Aspidites melanocephalus*).

**Figure 10:**
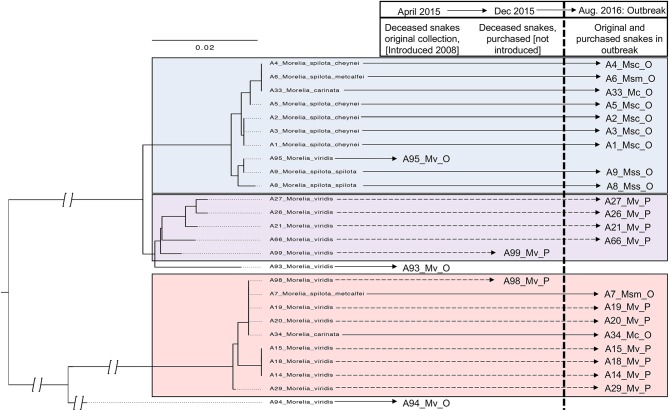
Phylogeny and proposed horizontal transmission in collection A. Nucleotide sequences (467 basepairs) aligned and analyzed with PhyML with HKY85 substitution model and 1,000 bootstrap replicates; arbitrary rooting; discontinuous scale in branches closer to root indicated by branch breaks. Snake ID denoted by A and number; Mv, *Morelia viridis*; Msc, *Morelia spilota cheynei*; Msm, *Morelia spilota metcalfei*; Mc, *Morelia carinata*. O, original collection. P, purchased.

Forty pythons in collection A were sampled longitudinally at approximately 4-month intervals over 28 months following an outbreak of serpentovirus that began in 2016. The infection status and clinical progression of individual snakes (Figure 9), and horizontal transmission (Figure 10) were assessed. Three snakes that were part of the original collection (introduced in 2008) were diagnosed with serpentovirus in 2015 and died (A93–95). Snakes A1–9, 30–31, and 33–36 were introduced between 2013 and 2015 and their infection status was unknown prior to the 2016 outbreak. Snakes A10–29 and 32 were purchased and introduced to the collection in 2016, after which the outbreak of serpentovirus-associated respiratory disease occurred. Snakes A98 and A99 were also purchased from the same seller and had contact with A10–29 and 32 but died prior to introduction into collection A. Positive snakes (tested at 0 and 4 months into the outbreak; A1–9 and 13–34) were quarantined away from remainder of collection. Snakes A37–40 were purchased after the outbreak (2017) and were never directly exposed to serpentovirus-positive snakes in the collection.

Overall, positive snakes remained positive during the 28-month period (Figure 9); no snakes were seen to transition from consistently positive to consistently negative. Snakes A11–12 (green tree pythons) were serpentovirus-negative at initial sampling were housed with the main collection and remained negative. Only one snake in the main collection converted to positive (A66); see “Vertical Transmission.” Two snakes (A35–36, olive pythons) that were initially negative, but potentially exposed to serpentovirus, were housed in the quarantine area; these snakes remained negative throughout the study. In a subset of quarantined snakes (A13–18), intermittent negative results were obtained despite overall positivity. These negative results primarily, but not exclusively occurred in snakes exhibiting lower respiratory scores (0–1). Sanger sequencing of PCR amplicons before and after time points with negative results yielded identical viral sequences.

Phylogenetic analysis of viral sequences from snakes in collection A was performed on 26 of 40 positive snakes; 5 viral genotype groups were detected, three of which were present in the 2016 outbreak (Figure 10). The first viral genotype group was found in one of the initial snakes diagnosed with serpentovirus (A95) and was also detected in other snakes from the original collection (A1–6, 8–9, 33), but not in any purchased snakes. The second and third viral genotypes were found in the purchased snakes that died before introduction into the collection (A98 and 99). These genotypes were also detected in the purchased snakes that were introduced (A21, 26, 27, 66) as well as snakes from the original collection (A7 and 34), consistent with transmission from the purchased snakes to the original collection. Longer viral sequences for phylogenetic analysis were not collected from all positive snakes in the collection, therefore, the presence of other viral genotypes not represented in the phylogeny cannot be ruled out.

### Vertical Transmission

During the longitudinal sampling of collection A, two separate serpentovirus-positive mating pairs (green tree pythons A27 and A29; jungle carpet pythons A1 and A3) were bred and resulted in a clutch of eggs. The green tree python mating pair resulted in 17 eggs and 16 viable hatchlings which were sampled orally at 4-month intervals for 20 months; 7 of these were only tested once due to donation to a separate collection and loss to follow-up within the first 4 months. The egg shells and liquid contents (post hatching) were pooled (4 eggs per pool) and all were found to be serpentovirus RNA positive. All offspring were negative at each sampling time-point except one (A66) that became serpentovirus-positive between 8 and 12 months of age. Sanger sequencing was performed on oral/choanal swabs from the adult male, female, and positive offspring, and the pooled egg shell/contents. Viral sequences from the adult female and the egg shell/contents were >99% similar to each other but only 84% similar to the viral sequences detected in the adult male and positive offspring, which were >98% similar to each other. A second mating pair of jungle carpet pythons produced a clutch of 9 eggs and 2 viable offspring that were sampled twice during a 6-month period. Egg pools were again found to be positive but the offspring remained negative during the sampling period.

## Discussion

In this study, 639 snakes from 62 species were screened for infection with ophidian serpentoviruses. Prevalence of infection was highest in pythons (nearly 40%) and serpentoviruses were detected in pythons in the *Morelia, Python, Malayopython, Antaresia*, and *Aspidites* genera. This finding agrees with reports in the literature that many python species are susceptible to serpentovirus infection (4–6, 9, 10). Most positive pythons in this study were *Morelia* species. The veterinary community has anecdotally regarded these pythons as being more predisposed to respiratory infection. It is possible that this belief reflected an increased susceptibility and high prevalence of serpentovirus infection in captive *Morelia* species. It is important to note that 5 of the collections in this study were known to have serpentovirus infections, which could have biased the sample set to increase prevalence measures. Nonetheless, these findings highlight the high rate of infection that can occur in collections in which serpentoviruses have been introduced.

In contrast to a high prevalence in pythons, serpentoviruses were detected at a significantly lower rate in snakes from the *Boidae* and *Colubridae* families and not detected in any snakes from the *Lamprophiidae, Elapidae*, or *Viperidae* families. These findings could indicate a reduced susceptibility or resistance to infection in these species. However, small sample sizes and targeted PCR assays may have impacted these results. Combined, only 30 lamprophiids, elapids, and vipers were tested and all were negative. Additionally, only 8 boas and 1 colubrid were found to be serpentovirus-positive out of 79 and 116, respectively. Based on the genetic diversity of serpentoviruses described in this study, it is possible that the PCR assay used may not have detected more divergent serpentoviruses in some of these species. Overall, these conclusions regarding species susceptibility and species specificity in non-python snakes must therefore be interpreted with caution and PCR-based diagnostics may need to be updated to account for the newfound diversity of serpentoviruses in snakes.

What can be concluded from these results is that non-python snakes are potentially less susceptible or resistant to infection by serpentoviruses that readily infect pythons. Phylogenetic analysis revealed that the same python serpentoviruses can infect pythons from multiple genera. But these viruses were never found to infect boas and colubrids in the same collections that had been exposed to infected pythons. In contrast, the viruses found in boas and colubrids (and reticulated pythons) belonged to distinct evolutionary lineages from those found in pythons. Therefore, although python serpentoviruses can infect pythons of multiple genera, barriers to infection may be too great for them to infect boas or colubrids, and vice versa. Interestingly, evolutionarily distinct serpentoviruses found in reticulated pythons were more closely related to viruses from boas and colubrids and an evolutionarily distinct serpentovirus found in a Dumeril's boa was more closely related to viruses from pythons (non-reticulated). Sampling of wild-caught snakes will provide insight into the origins and natural hosts of these distinct serpentoviruses.

Clinical disease was consistently observed for all virus genotypes detected in the clade of python-associated serpentoviruses, whereas clinical disease was absent in boas, colubrids, and reticulated pythons (serpentoviruses outside the python-only clade). The virus detected in a Dumeril's boa was the only virus associated with clinical disease in a boa species. Its closer relation to python viruses may provide evidence that certain genotypic lineages are more likely to cause disease in snakes, or that certain snakes are more susceptible to disease with these viruses. However, this hypothesis will likely be altered as a greater number of serpentoviruses in non-python species or divergent serpentoviruses are discovered.

The single serpentovirus sequence found in a colubrid in the study (L25) was most closely related a sequenced recovered from a snake-associated nematode (1). Previously it was shown that serpentovirus is detectable in gastrointestinal tissue and feces in infected ball pythons (13) and it is hypothesized that these putative worm viruses (1) actually represent snake viruses that were ingested by or contaminated the surface of intestinal nematodes.

Previous studies evaluating the prevalence of viral infections or respiratory disease in pythons and boas found a greater prevalence in pythons, as well as correlations with older age and specific husbandry practices (9, 19, 20). Similarly, the study found older snakes were more likely to be infected, but increasing age did not increase the likelihood of clinical disease. Therefore, infection in older animals is suspected to be due to increasing time of potential exposure, rather than physiologic changes that accompany age. Husbandry was not directly assessed in this study but it is a significant variable in the health of captive reptiles and suboptimal practices can result in stress and alterations in immunity (21, 22). If one can assume similar husbandry practices within a collection, it would be expected that snakes of the same species infected with the same or highly similar viruses would exhibit similar degrees of clinical disease. In some collections, this trend was observed (e.g., Collection H), whereas in others, clinical disease was more variable (e.g., Collection A). Beyond husbandry, individual animal factors that could have played a role in disease variability include length of time infected and exposure dose, co-morbidities (both infectious and non-infectious), reproductive stage/status, and genetic background of the host (23). Other inciting stress events could have also affected groups of snakes within a collection, including seasonal or environmental change, relocation/shipping, breeding, and increased handling or showing (23).

Coinfections have been documented in natural infections of viruses related to serpentoviruses, such as toroviruses and coronaviruses, and have been associated with viral recombination and evolution (24, 25). The snake in which serpentovirus coinfection was detected (H0) died from severe respiratory disease, but it is unknown if coinfection altered the disease course or pathologic progression. Coinfections with other known and unknown non-serpentovirus snake pathogens may also have played roles in disease progression. Other agents including paramyxoviruses, orthoreoviruses, sunshine virus, and *Mycoplasma* species have been associated with, or shown to cause respiratory disease in snakes (20, 26–28). The ecological interactions of serpentoviruses with other pathogens and the host immune system merits further study.

One of the primary goals of this project was to assess aspects of disease that may lend themselves to better management strategies including quarantine practices, the potential for viral clearance, the frequency of diagnostic testing, and the risk of transmission to offspring following breeding.

In two collections a significantly lower rate of infection in the main population compared to infected snakes in quarantine was observed. Collection A and H removed infected snakes and snakes that had been in direct or close contact with infected snakes, from the main collection and placed them in separate quarantine buildings. A single designated caretaker was assigned, either indefinitely or each day, for quarantine management. Quarantine-specific clothes and equipment, separate air-flow and ventilation, and rigorous disinfection practices of the facility were utilized (see prevalence results). In both cases, infection rates were high in quarantined snakes (68–100%) and remained low (<5%) in the main collection. These findings indicate that transmission occurs at a higher rate between snakes of close-proximity and that proper quarantine practices that limit the spread of infectious agents can be highly effective. Possible routes of transmission are speculated to include fecal-oral or respiratory fomite transmission or direct contact during breeding events, which have the potential to be limited or eliminated through quarantine measures (13).

Phylogenetic analysis of a single collection (A) over time revealed that existing infected snakes and introduced infected snakes could both contribute to overall serpentovirus burden. Furthermore, longitudinal testing of this same collection revealed that serpentovirus infection can persist over long periods and infection can result in overt or subclinical disease. In this study, intermittent negative results in a low number of snakes were initially thought to represent viral clearance, but sampling of these snakes over time revealed continued evidence of infection. Negative PCR results could represent episodes of viral clearance followed by re-infection or periods of reduced shedding below the limit of detection. No snakes were observed to transition from consistently positive to consistently negative test results, indicating complete clearance may not be possible. Technical factors could also play a role in “false-negative” results, such as insufficient swabbing or sample handling. Independent of the underlying cause of intermittent negative results, the chronicity of disease and the potential for negative test results in infected snakes highlights two important management strategies: the need for longer periods of quarantine prior to the introduction of potentially infected snakes (at least 3 months) and the requirement of multiple negative diagnostic tests over time to ensure true serpentovirus-negativity in individual snakes.

Also in collection A, infected snakes that exhibited moderate to severe respiratory signs were treated with antibiotics (enrofloxacin and ceftazidime) following bacterial culture of sterile lung washes that yielded heavy growth of *Pseudomonas* species and moderate growth of *Escherichia coli* and *Citrobacter* species. Additionally, subcutaneous lactated ringers fluid therapy and an increased thermal gradient were provided. Secondary bacterial infections have been observed in association with serpentovirus infection (4, 6, 13). In some cases, treated snakes exhibiting moderate to severe clinical signs lived up to 16 months post-diagnosis, which could be associated with antibiotic treatment and supportive therapy. Although this observation was only anecdotal and not a monitored aspect of this study, it is possible that treatment of sequelae could contribute to prolonged lifespan in persistently serpentovirus-infected collections.

Vertical transmission is a natural route of spread for some nidoviruses (e.g., equine arteritis virus, gill-associated virus) (29, 30). In this study, vertical transmission was evaluated in the eggs and offspring of serpentovirus-positive mating pairs. Viral RNA was detected in/on most eggs, which was suspected to represent contamination from feces within the cloaca rather than infection of the embryo or egg contents. In offspring, all but one remained negative following hatching (1/19). In the one positive offspring, the viral genotype was more closely related to the male parent than the female parent. Therefore, these findings may support vertical transmission of serpentoviruses from the male, but at a significantly lower rate than horizontal transmission. Furthermore, in this study eggs were artificially incubated and sterilized with UV or a quaternary ammonium compound and hatchlings were housed separately from other infected snakes. The prevalence of infection in offspring hatched under natural conditions was not examined, but could provide insight into the likelihood of horizontal transmission from parent to offspring under more typical conditions. Lastly, a low rate of hatching was observed in one clutch (2/9), which was suspected to be due to artificial incubation errors, but this cannot be confirmed.

Serpentoviruses are significant respiratory pathogens of pythons and introduction into collections can be devastating. Due to the chronicity and variability in clinical signs during infection with serpentoviruses, stringent management strategies are recommended. These include prolonged quarantine practices with multiple negative test results prior to the introduction of new snakes into the collection, the immediate removal or separation of any positive snakes from the main collection with strict quarantine measures to prevent continued spread, and understanding the risk of horizontal and vertical transmission when purchasing, selling, or breeding snakes. The clinical importance of serpentovirus infection in boas and colubrids remains poorly understood, but infection is possible and should be perceived as a potential respiratory pathogen. Serpentovirus infection in other snake species continues to be an ongoing field of research.

## Data Availability Statement

The datasets generated for this study can be found in the GenBank and BioProject/BioSample/Short Read Archive.

## Ethics Statement

Ethical review and approval was not required for the animal study because non-invasive samples were collected by owners and submitted to our laboratory. No animal handling, manipulation, or sampling was performed in our laboratory on live animals.

## Author Contributions

LH-H was responsible for performing experiments, analyzing and interpreting the data, and drafting of the manuscript. RO, PB, SF, SP, AS, and EJ provided samples and collected epidemiologic data for the study. JW, ED, and RO provided primers and protocols for the study. SC and MS performed metagenomic sequencing and data analysis. All authors contributed to manuscript revision, read, and approved the submitted version.

### Conflict of Interest

PB is a co-founder and owner of Terrestrial & Arboreal. SF is the founder and owner of Fishhead Labs. The remaining authors declare that the research was conducted in the absence of any commercial or financial relationships that could be construed as a potential conflict of interest. The handling editor declared a past co-authorship with one of the authors RO.
